# Determinants for non-sentinel node metastases in primary invasive breast cancer: a population-based cohort study of 602 consecutive patients with sentinel node metastases

**DOI:** 10.1186/s12885-019-5823-x

**Published:** 2019-06-25

**Authors:** Shabaz Majid, Lisa Rydén, Jonas Manjer

**Affiliations:** 1 Department of Surgery, Central Hospital of Kristianstad, SE-291 85 Kristianstad, Sweden; 2 Department of Clinical Sciences Malmö, Lund University, Malmö, Sweden; 3 Department of Surgery, Skåne University Hospital, Malmö, Sweden; 4 Department of Clinical Sciences Lund, Lund University, Lund, Sweden

**Keywords:** Invasive breast cancer, Sentinel node metastases, Non-sentinel node metastases, Determinants, Completion axillary lymph node dissection

## Abstract

**Background:**

Sentinel node biopsy (SNB) is the standard procedure for axillary staging in patients with clinically lymph node negative invasive breast cancer. Completion axillary lymph node dissection (c-ALND) may not be necessary for all patients as a significant number of patients have no further metastases in non-sentinel nodes (non-SN) and c-ALND may not improve survival. The first aim of our study is to identify clinicopathological determinants associated with non-SN metastases. The second aim is to determine the impact of the number of sentinel node (SN) with macro-metastases and the type of SN metastases on metastatic involvement in non-SN.

**Methods:**

This is a retrospective study of 602 patients with primary invasive breast cancer operated on with SNB and c-ALND in Lund and Malmö during 2008–2013. All these patients had micro- and/or macro-metastases in SNs. Information was retrieved from the national Information Network for Cancer Care (INCA). The risk of metastases to non-SNs were analyzed in relation to clinicopathological determinants such as age, screening mammography, tumour size, tumour type, histological grade, estrogen status, progesterone status, HER2 status, multifocality and lymphovascular invasion. Additionally, we compared the association between the number of the SN and the type of metastases in SN with the risk of metastases to non-SNs. Binary logistic regression was used, yielding odds ratios (OR) with 95% confidence intervals (CI).

**Results:**

We found that 211 patients (35%) had metastases in non-SNs and 391 patients (65%) had no metastases in non-SNs. Lobular type (18%) of breast cancer (1.73; 1.0 1-2.97) and multifocal (31.3%) tumours (2.20; 1.41–3.44) had a high risk of non-SNs metastases. As compared to only micro-metastases, the presence of macro-metastases in SNs was associated with a high risk of metastases to non-SNs (4.91; 3.01–8.05). The number of SN with macro-metastases, regardless of the number of SNs removed by surgery, increases the risk of finding non-SNs with metastases. The total number of SN removed by surgery had no impact on diagnosis of metastases in non-SNs. No statistically significant associations were observed regarding other studied determinants.

**Conclusion:**

We conclude in the present study that lobular cancer and multifocal tumours were associated with a high risk of non-SN involvement. The presence of the macro-metastases in SNs and the number of SN with macro-metastases has a positive association with presence of metastases in non-SNs. The total number of SNs removed by surgery had no impact on finding metastases in non-SNs. These factors may be valuable considering whether or not to omit c-ALND.

## Background

Sentinel node biopsy (SNB) is the standard procedure for axillary staging in patients with clinically lymph node negative breast cancer. Completion axillary lymph node dissection (c-ALND) has traditionally been performed at many breast centres when the final pathological report reveals macro-metastases in 1–2 sentinel lymph nodes [1, 2]. However different studies, including Z0011 trial from American College of Surgeons Oncology Group (ACOSOG), show that c-ALND is not contributed to better survival [3, 4]. Still axillary lymph node status remains one of the most important and powerful prognostic factor in invasive breast cancer as it predicts clinical outcome and is an indication for systemic therapy [5, 6].

SN metastases are classified according to the size of metastases; isolated tumour cells (ITC < 0.2 mm), micro-metastases (0. 2-2.0 mm) and macro-metastases (> 2.0 mm) [7]. In our department, and still in many other centers, the main indication to perform a c-ALND is involvement of SN with macro-metastases. The presence of ITC or micro-metastases is no longer an indication to perform a c-ALND when the patient is planned to undergo radiation therapy, e.g. in breast conserving surgery [8].

There are many benefits of SNB such as avoidance of unnecessary ALND in patients with no axillary metastases*.* Most of the complications associated with ALND might also occur after SNB. However, the risk of developing bleeding or infection post operatively is less likely to occur following SNB as compared to ALND. Moreover, the incidence of developing pain, sensory disorder or lymph oedema in the upper arm is very low after SNB [9]. Still the SNB as a procedure is time consuming and needs resources.

It is still unknown if c-ALND is necessary to be performed in all cases with metastatic involvement of SN and the possibility of omitting c-ALND has been discussed in several studies, as the risk of metastases to non-SNs may be low and the impact of an ALND on survival is not clear [3, 4].

The first aim of our study is to identify clinicopathological determinants associated with metastases to non-SNs in patients with metastases in SNs. The second aim is to determine the impact of the number of SN with macro-metastases and the type of SN metastases on metastatic involvement in non-SNs.

## Methods

The Information Networks for Cancer (INCA) is a nationwide database for breast cancer in Sweden which is available on an IT platform. This registry collects information about the cancer care and manages long term follow up. The center in Southern Sweden which manages the registry is the Regional Cancer Center in Southern Sweden (RCC-Syd). By law, all cancer diagnoses have to be reported to the Swedish Cancer Registry and this routine is implemented through INCA.

In this study we included all women operated on because of breast cancer in Lund and Malmö during the period of January, 1st 2008 to December, 31st 2013. They were identified using the clinical registry INCA, and a total number of 3979 cases with breast cancer were found using the unique twelve-digit Swedish civil registration number.

**Fig. 1:**
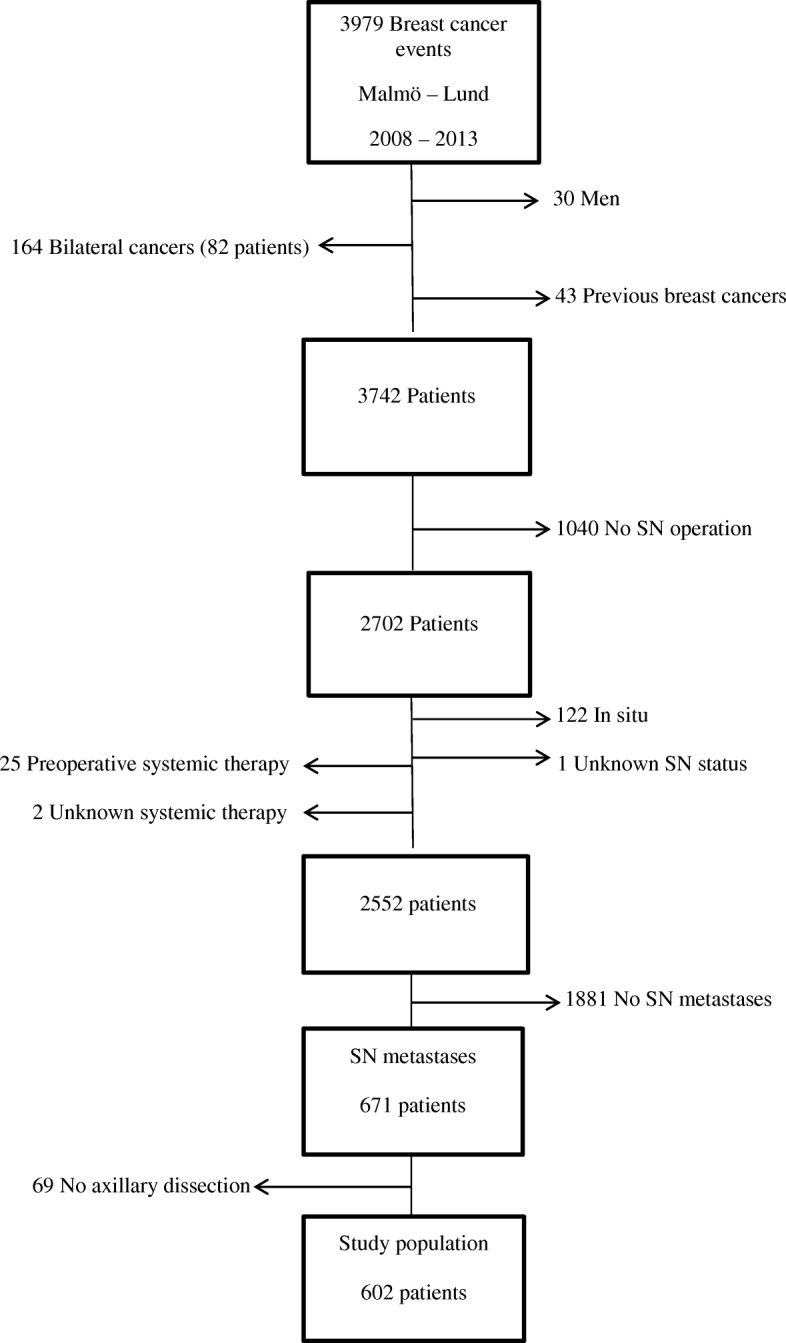
Patient selection

We excluded the following patients from the main study population; 43 cases with previous breast cancer, 122 cases with in situ breast cancer, 82 cases with bilateral breast cancer, 1040 cases who were not operated on with SNB and 25 patients who had received neoadjuvant systemic therapy. There were two patients who had unknown information about the systemic therapy and one patient had unknown status about SN surgery, finally all 30 male patients were excluded in this analysis. Among all patients in the study population there were 1881 patients who had no metastases in SN. We identify totally 671 cases with SN metastases including 69 women who did not undergo a subsequent ALND. The final study population following these exclusions resulted in 602 cases with metastases in SN and all these cases were operated on with c-ALND (Fig. 1).

This study was approved by the regional ethical review board of Lund University (reference 2013/821).

All included patients in this study have been reviewed and discussed at a multidisciplinary breast cancer conference (surgery, radiology, oncology and pathology) at Skåne University Hospital in Malmö and Lund. INCA has a unique and specially designed registration form and all available information about every breast cancer case transfers to the INCA platform. In the present study we retrieved information from INCA about SLNB and c-ALND, i.e. number of lymph nodes removed, type of metastases as well as information about histopathological type and grade, receptor status, HER2 status, tumour size, multifocality, lymphovascular invasion, age and menopause status.

The mode of cancer detection was recorded as screening-detected vs. not detected by screening mammography. We identify menopause status as pre- or post-menopausal. The post-menopausal women were further more sub-classified according to their last menstruation, i.e. 6 months to 5 years or more than 5 years after menopause.

WHO-classification system has been used to identify the histopathological types, accordingly six types of invasive cancer were identified [10–12]. Furthermore these six types were merged into four different groups i.e. ductal, lobular, combined ductal with lobular and other rare types. Nottingham histological grading score (NHG) was used to define the histological grades [13, 14]. TNM classifications were used to define the tumour size. T1 tumour _≤_ 20 mm, T2 tumour 21-50 mm and T3-T4 > 50 mm [10]. Lymphovascular invasion was defined according to the Swedish society of pathology (KVAST) classification, i.e. invasion of vessel wall, underlying endothelium or vascular spaces by tumour cells.Two or more tumours with normal tissue and/or in situ tumours at a distance of 20 mm were regarded as multifocal tumours [15]. Estrogen and progesterone were measured by immunohistochemistry (IHC), and a positive receptor status was identified when the receptor percentage was more than 10% while receptor status was regarded as negative when the percentage was less than 10% [7]. IHC was used to analyze HER2 protein and test results 1+, 2+, or 3+ were reported. IHC test was completed by Fluorescent In Situ Hybridization (FISH) in cases where HER2 was 2+ or 3+. HER2 status was classified as negative when HER2 IHC =0–1, 2+ or 3+ in non-amplified tumours. All cases of HER2 2+ or 3+, which were amplified by FISH, regarded as positive HER2 tumours [16].

In the present analysis metastases in non-SNs were regarded as macro metastases when the size was > 2 mm and as micro-metastases when the size was 0. 2-2.0 mm. Metastases with a size less than 0.2 mm were regarded as isolated tumour cells (ITC). All ITC regarded as no metastases according to international guideline for lymph node metastases [7]. Non-SNs with macro- or micro-metastases were regarded as positive, and those without metastases as negative.

We used binary logistic regression to compare the association between different determinants and metastases in non-SNs. We adjusted all analyses for all studied determinants i.e. histopathological type and grade, presence of multifocality, presence of lymphovascular invasion, receptor status for estrogen and progesterone, HER2 status, tumour size, menstrual status, screening and age. Binary logistic regression was also used to compare the association between the number of the SN, the type of metastases in SN and the risk of metastases to non-SNs. These analyses included a limited number of events and only a selected set of co-variates were included in the multivariate analysis, i.e. those statistically significantly associated with metastases in non-SNs (screening, tumour types and multifocality). This analysis was also stratified for the number of SLNs which had been removed. Odds ratios (OR) with 95% confidence intervals (CI) were analyzed. For all analyses we used the Statistical Package for the Social Sciences (SPSS) program version 22.0 (SPSS Institute, Chicago, IL, USA).

## Results

Out of the 602 patients operated on with c-ALND, 211 patients (35%) had metastases in non-SNs and 391 patients (65%) had no metastases in non-SNs, Table 1. There was a high risk of metastases to non-SNs in women with lobular type tumour (18%) compared with ductal type tumours (1.73; 1.01–2.97). Multifocal tumour (31.3%) were also associated with a high risk of non-SN metastases compared with unifocal tumours (2.20; 1.41–3.44), Table 2. There was a high risk for non-SN metastases in 11 patients with unknown status for mammography screening as mode of detection, compared to those patients who were not diagnosed by screening mammography (4.70; 1.36–16.19), Table 2. There were no other statistically significant associations between all other studied determinants and involvement of non-SNs, Table 2.

**Table 1:** Potential determinants in relation to non-sentinel node status

Determinants	Category	Total	Negative Non-SN		Positive Non-SN	
			N	%	N	%
Screening	No	309	197	50.4	112	53.1
	Yes	278	190	48.6	88	41.7
	Unknown	15	4	1.0	11	5.2
Age	≤50	151	103	26.3	48	22.7
	51–74	370	239	61.1	131	62.1
	≥75	81	49	12.5	32	15.2
Menopause Status	Pre	153	104	26.6	49	23.2
	Post < 5 ys	57	42	10.7	15	7.1
	Post ≥5 ys	370	231	59.1	139	65.9
	Unknown	22	14	3.6	8	3.8
Tumour size	T1	331	220	56.3	111	52.6
	T2	193	120	30.7	73	34.6
	T3 & T4	10	6	1.5	4	1.9
	Unknown	68	45	11.5	23	10.9
Tumour type	Ductal	490	331	84.7	159	75.4
	D & L	14	5	1.3	9	4.3
	Lobular	79	41	10.5	38	18.0
	Other	19	14	3.6	5	2.4
Histological grade	I	118	82	21.0	36	17.1
	II	272	176	45.0	96	45.5
	III	210	132	33.8	78	37.0
	Unknown	2	1	0.3	1	0.5
Estrogen receptor	Positive	545	358	91.6	187	88.6
	Negative	56	33	8.4	23	10.9
	Unknown	1	0	0.0	1	0.5
Progesterone receptor	Positive	478	319	81.6	159	75.4
	Negative	122	72	18.4	50	23.7
	Unknown	2	0	0.0	2	0.9
HER2 status	Negative	383	248	63.4	135	64.0
	Positive	61	33	8.4	28	13.3
	Unknown	158	110	28.1	48	22.7
Multifocality	No	355	247	63.2	108	51.2
	Yes	129	63	16.1	66	31.3
	Unknown	118	81	20.7	37	17.5
Vascular invasion	No	241	166	42.5	75	35.5
	Yes	91	60	15.3	31	14.7
	Unknown	270	165	42.2	105	49.8

**Table 2:** Potential determinants for non-sentinel node metastases

Determinants	Category	Negative Non-SN	Positive Non-SN	OR 95% CI	OR 95% CI^a^
Screening	No	197	112	1.00	1.00
	Yes	190	88	0.81 (0.58–1.15)	0.81 (0.54–1.21)
	Unknown	4	11	4.84 (1.50–15.55)	4.70 (1.36–16.19)
Age	≤50	103	48	1.00	1.00
	51–74	239	131	1.18 (0.79–1.76)	1.50 (0.53–2.06)
	≥75	49	32	1.40 (0.80–2.46)	1.08 (0.45–2.60)
Menopause Status	Pre	104	49	1.00	1.00
	Post <5ys	42	15	0.76 (0.38–1.50)	0.79 (0.34–1.86)
	Post ≥5ys	231	139	1.28 (0.86–1.90)	1.21 (0.60–2.44)
	Unknown	14	8	1.21 (0.48–3.08)	1.45 (0.52–4.05)
Tumour size	T1	220	111	1.00	1.00
	T2	120	73	1.21 (0.83–1.74)	1.11 (0.74–1.66)
	T3 & T4	6	4	1.32 (0.36–4.78)	0.78 (0.19–3.14)
	Unknown	45	23	1.01 (0.58–1.76)	0.76 (0.40–1.44)
Tumour type	Ductal	331	159	1.00	1.00
	D & L	5	9	3.75 (1.24–11.36)	2.93 (0.92–9.37)
	Lobular	41	38	1.93 (1. 19-3.12)	1.73 (1.01–2.97)
	Others	14	5	0.74 (0. 26-2.10)	0.85 (0. 29-2.50)
Histological grade	I	82	36	1.00	1.00
	II	176	96	1.24 (0.78–1.98)	0.88 (0.53–1.46)
	III	132	78	1.35 (0.83–2.18)	0.94 (0.54–1.65)
	Unknown	1	1	2.28 (0. 14-37.43)	1.23 (0.07–21.34)
Estrogen receptor	Positive	358	187	1.00	1.00
	Negative	33	23	1.33 (0.76–2.34)	1.04 (0.47–2.34)
	Unknown	0	1	–	–
Progesterone receptor	Positive	319	159	1.00	1.00
	Negative	72	50	1.40 (0.93–2.09)	1.17 (0.66–2.07)
	Unknown	0	2	–	–
Her-2 status	Negative	248	135	1.00	1.00
	Positive	33	28	1.56 (0.90–2.69)	1.52 (0.82–2.82)
	Unknown	110	48	0.80 (0.54–1.19)	0.88 (0.55–1.39)
Multifocality	No	247	108	1.00	1.00
	Yes	63	66	2.40 (1.59–3.62)	2.20 (1.41–3.44)
	Unknown	81	37	1.04 (0.67–1.64)	0.99 (0.61–1.60)
Vascular invasion	No	166	75	1.00	1.00
	Yes	60	31	1.14 (0.68–1.91)	1.13 (0.64–1.98)
	Unknown	165	105	1.41 (0.98–2.03)	1.31 (0.86–1.99)

^a^Adjusted for screening, age, menopause status, tumour size, tumour type, histological grade, estrogen status, progesterone status, HER2 status, multifocality, lymphovascular invasion

The total number of SNs removed by surgery had no clear impact on finding metastases in non-SNs, Table 3. The presence of macro-metastases in SN was associated with a high risk of metastases to non-SNs compared with presence of only micro-metastases in SNs (4.91; 3.01–8.05), Table 3. Stratified analysis showed that the number of SNs with macro-metastases, regardless the number of SNs removed by surgery, increases the risk of finding non-SNs with metastases. Combined analysis using one SN with only micro-metastases as reference showed a positive correlation between the number of SNs with macro-metastases and the possibility of non-SN involvement with metastases, Table 4.

**Table 3:** Number and type of metastases in sentinel node and risk of metastases in non-sentinel node

SN	Category	Total (n)	Negative Non-SN (n)	Positive Non-SN (n)	Positive Non-SN (%)	OR (95% CI)	OR^a^ (95% CI)
SN removed (n)	1	118	84	34	28.8	1.00	1.00
	2	208	125	83	39.9	1.64 (1.01–2.66)	1.34 (0.77–2.31)
	3	166	110	56	33.7	1.26 (0.75–2.10)	1.08 (0.61–1.93)
	4	83	56	27	32.5	1.19 (0.65–2.19)	0.96 (0.48–1.90)
	≥5	25	15	10	40.0	1.65 (0.67–4.03)	1.71 (0.65–4.53)
	Unknown	2	1	1	–	–	–
	Total	602	391	211			
Type of metastases in SN^b^	Micro	186	159	27	14.5	1.00	1.00
	Macro	414	232	182	43.9	4.62 (2.94–7.26)	4.91 (3.01–8.05)
	Unknown	2	0	2	–	–	–
	Total	602	391	211			

^a^Adjusted for screening, age, menopause, tumour size, tumour type, histological grade, estrogen receptors, progesterone receptors, HER2, multifocality and lymphovascular invasion

^b^If both micro- and macro-metastases, classified as macro-metastases

**Table 4:** Number of macrometastases in sentinel node and risk of metastases in non-sentinel nodes

SN removed	Macro- metastases	Total	Negative Non-SN	Positive Non-SN	Positive Non-SN	Stratified analysis		Combined analysis	
(n)	(n)		(n)	(n)	(%)	OR (95% CI)	OR (95% CI)^a^	OR (95% CI)	OR (95% CI)^a^
1	0	47	39	8	17.0	1.00	1.00	1.00	1.00
	1	69	44	25	36.2	2.77 (1. 12-6.85)	2.65 (1.05–6.66)	2.77 (1. 12-6.85)	2.65 (1.05–6.66)
	Unknown	2	1	1	–	–	–	–	–
2	0	51	45	6	11.7	1.00	1.00	0.65 (0. 21-2.04)	0.65 (0. 21-2.07)
	1	105	60	45	42.8	5.62 (2. 21-14.33)	4.83 (1.87–12.49)	3.66 (1.56–8.58)	3.09 (1.30–7.39)
	2	52	20	32	61.5	12.00 (4.33–33.23)	11.12 (3.97–31.19)	7.80 (3.03–20.04)	7.43 (2.83–19.50)
3	0	59	51	8	13.5	1.00	1.00	0.76 (0. 26-2.22)	0.68 (0. 23-2.02)
	1	58	39	19	32.7	3.11 (1. 23-7.83)	3.68 (1.32–10.24)	2.37 (0.93–6.07)	2.15 (0.82–5.64)
	2	28	14	14	50.0	6.37 (2. 23-18.23)	6.30 (1.99–19.99)	4.87 (1.69–14.10)	4.18 (1.40–12.50)
	3	21	6	15	71.4	15.94 (4.77–53.18)	16.96 (4.42–65.12)	12.19 (3.62–41.05)	10.02 (2.89–34.81)
4	0	24	21	3	12.5	1.00	1.00	0.70 (0. 17-2.91)	0.57 (0. 13-2.49)
	1	29	22	7	24.1	2.23 (0.51–9.77)	3.34 (0.55–20.15)	1.55 (0.50–4.85)	1.56 (0.49–4.94)
	2	11	7	4	36.3	4.00 (0.71–22.43)	9.46 (1. 26-70.85)	2.79 (0.66–11.82)	2.92 (0.67–12.65)
	3	14	4	10	71.4	17.50 (3. 28-93.49)	17.18 (2.34–126.2)	12.19 (3.04–48.77)	9.25 (2. 22-38.53)
	4	5	2	3	60.0	–	–	*–*	–

Stratified analysis; comparisons within groups defined by number of removed SNs. Combined analysis; all groups compared using one SN with only micro-metastases as reference

^a^Stratified and combined analysis adjusted for screening, tumour type, and multifocality

## Discussion

The present registry-based study showed that 65% of patients, who underwent c-ALND because of SN metastases, have no further additional non-SN involvement. Lobular types (18%) and multifocal tumours (31.3%) were associated with a high risk of non-SN metastases. The total number of SN removed by surgery had no impact on finding metastases in non-SNs. On the contrary the presence of macro-metastases in SNs contributed with higher risk of metastases to non-SNs. The number of SN with macro-metastases is also associated with the higher risk of finding non-SNs with metastases. Furthermore there was a positive association between the number of SN with macro-metastases and the probability of non-SNs involvement with metastases regardless the number of SNs removed by surgery.

Axillary lymph node status is an important factor in managing patients with primary breast cancer. SNB is the standard method for staging, however the value of the c-ALND has been questioned during the last decade as the majority of these patients have disease-free non-SNs and omitting c-ALND probably has no impact on survival [3].

This analysis included 602 patients from a non-selected population-based cohort of consecutive cases with essential data available from the main breast cancer registry in southern Sweden (INCA) which is a strength of our study. A limitation is however, that we had no information on why 69 women who had a positive SNB did not undergo a c-ALND. Furthermore, analysis based on the data collected from a registry and the reliability of collected data might be questioned, however the quality of the INCA registry is regarded as very high with periodic validation control [17]. A potential problem is, however, that the availability of information about different clinicopathological determinants used in the present study might be limited or unavailable preoperatively, before the final pathological results are available, and this may limit the pre-operative value of these determinants. Previous studies have suggested that the internal mammary lymph node status is an independent prognostic factor. A limitation of the present analysis is that there was no information on internal mammary lymph nodes in the INCA data base. However, this information is not used in clinical practice and currently has no impact on treatment.

In the present analysis we found that 65% of patients, underwent c-ALND because of SN metastases, have no further additional non-SN metastases, this may suggest the possibility of omitting ALND in certain cases with SN metastases but this demands accurate identification of low risk patients. Different studies have questioned the value of c-ALND even if there are metastases in the SN. The Z0011 randomized trial from the American College of surgeons Oncology Group (ACOSOG) compared ALND versus no axillary surgery in patients with a maximum of two SNs with metastases, and the study supported the view that there is no negative impact on survival for patients where an ALND is omitted [4].

Our study showed that there was a high risk of metastases to non-SNs in patients with lobular type compared with ductal type tumours. Adachi Y. et al. showed in their study including 3771 patients that 31 cases with lobular type (18%) had more non-SN metastases than 457 (21%) cases with ductal type and lobular cancer was an important factor for the prediction of non-SN positivity in cases with macro-metastases in SNs. Adachi Y. et al. thus suggested that omitting c-ALND for lobular type with positive SNs requires more consideration [18]. Previous studies showed that loss of E-Cadherin in the extra cellular space and the differences in gene expression between lobular and ductal cancers are associated with immune response, cell invasion and cell adhesion which might be a possible reason for metastatic involvement of lymph nodes in lobular type of breast cancer [19].

We observed in our study that mode of detection (screening mammography vs not) had no clear impact on finding non-SN metastases. Tvedskov et al. showed in their study involving 995 patients, registered in the Danish Breast Cancer Cooperative Group (DBCG) Database, that there was no large difference in the risk of non-SN metastases between patients with clinically detected and screening detected cancers with micro-metastases or ITC in the SN [20]. In our study there was a high risk for non-SN metastases in 11 patients with unknown status for mode of detection. This may be a chance finding, but we choose to include this variable in the multivariate analyses for type and number of SN metastases.

In this cohort we observed that multifocal tumour were associated with high risk of non-SN involvement with metastases. Similarly Cabioglu et al. found in their study including 1322 patients with invasive breast cancer that multifocal tumour had more potentials of metastases to axillary lymph nodes compared with unifocal invasive tumour, regardless of primary tumour size. It is unclear with underlying biology regarding the multifocality and increased risk of lymph node involvement but the aggressiveness of multifocal tumours has been proposed as underlying cause in some studies, another proposed theory is finding higher proportion of lobular type in multifocal tumours compared with unifocal tumour. Furthermore using the largest diameter or the combined diameter of the multifocal tumors, as the size of the tumour, has been proposed as a possible explanation [21].

There were no statistically significant findings for other determinants included in this study i.e. age, menopause status, tumour size, histological grade, estrogen status, progesterone status, HER2 status, lymphovascular invasion. Y. Andersson et al. showed in their analysis that tumour size and histological grade were significantly associated with non-SN status [22]. Dighe L. et al. showed in their study that tumour size and vascular invasion were strongly associated with the metastatic involvement of SN, and they created a nomogram that facilitate preoperative decision-making regarding the extent of axillary surgery [23]. The use of nomograms has also been suggested by others, and some are available as a web-based tool [24]. A metanalysis performed by van la Parra RF. et al. included data from 56 candidate studies showed that eight different variables possibly related to high risk of finding non-SN metastases. These 8 individual characteristics were; size of metastases in the SN, extracapsular extension in the SN, number of the positive SN, number of the negative SN, tumour size, ratio of positive sentinel nodes, lymphovascular invasion in the primary tumour and method of detection, all these predictors were associated with high risk of finding metastases in non-SNs [25].

In this analysis we observed that the total number of SN removed by surgery has no impact on finding metastases in non-SNs, while the type of metastases in SN is an important predictor for non-SN metastases where presence of macro-metastases in SN strongly contributed with a high risk of finding additional non-SN involvement with metastases compared with presence of micro-metastases in SN. Van den Hoven I. et al. showed in their analysis including 513 patients with positive SN underwent c-ALND at 10 participating hospitals that the presence of negative SN as well as continuous size of the largest SLN metastases are strong predictors for the presence of metastases in the non-SNs [26]. Similarly, Elisabeth A. Mittendorf et al. and Hwang RF. et al. have observed in their studies that the size of metastases in the SN was the most important predicting variable for the presence of additional non-SN involvement [27, 28].

We also found that not only the type of metastases has a positive association with the risk of non-SN metastases but the number of SN with macro-metastases was associated with the risk of metastases in non-SNs regardless of the total number of SNs removed at surgery. Combined analysis, using one SN with only micro-metastases as a reference, showed a positive correlation between the number of SN with macro-metastases and the risk of non-SN involvement with metastases. Siem A. Dingemans et al. showed in their analysis that in patients with macro-metastases in SNs, tumor size larger than 2 cm, extranodal growth, and non-negative SNs are predictors of non-SN involvement [29].

The present study provides evidence that clinicopathological determinants such as lobular type or multifocality as well as the type of SN metastases and the number of the SN with macro-metastases may possibly be used as supporting tools in evaluating the risk of lymphatic spread to the non-SNs and may help clinician in taking final decision before performing c-ALND, however the benefit of the c-ALND, even when there are macro-metastases in the non-SNs, is not clear and an accurate identification of the low risk patients who may possibly omit c-ALND is still difficult.

## Conclusion

We conclude that lobular cancer and multifocal tumours are associated with a high risk of non-SN involvement. The presence of the macro-metastases in SNs, vs. only micro-metastases, and the number of SN with macro-metastases has a positive association with metastases in non-SNs. These factors may be valuable considering whether or not to omit c-ALND.

## Data Availability

The data that support the findings of this study are available from INCA but restrictions apply to the availability of these data, which were used under license for the current study, and so are not publicly available. Data are however available from the authors upon reasonable request and with permission of INCA.

## Abbreviations

ACOSOG: American College of Surgeons Oncology Group
ALND: Axillary Lymph Node Dissection
c-ALND: Completion Axillary Dissection
CI: Confidence Interval
FISH: Fluorescent in situ hybridization
HER2: Human Epidermal Growth Factor Receptor 2
IHC: Immunohistochemistry
INCA: Information Network for Cancer Care
ITC: Isolated Tumour Cell
KVAST: Swedish society of pathology
NHG: Nottingham Histological Grading
non-SN: Non-Sentinel Node
OR: Odds Ratio
RCC-Syd: Regional Cancer Center in southern Sweden
SN: Sentinel Node
SNB: Sentinel Node Biopsy
SNM: Sentinel Node Metastases
SPSS: Statistical Package for the Social Sciences
TNM: Tumour Lymph Node Metastasis
WHO: World Health Organization
